# Supplementary material for: Drug induced liver injury is associated with high mortality—A study from a tertiary care hospital in Pakistan
Source: PLoS One. 2020 Apr 10;15(4):e0231398. doi: 10.1371/journal.pone.0231398 (PMC7148123; doi:10.1371/journal.pone.0231398)
Supplement: S1 Data — (RTF) [file pone.0231398.s001.rtf]

Frequencies
DILI_severity	
	Frequency	Percent	Valid Percent	Cumulative Percent	
Valid	Mild	204	44.2	44.2	44.2	
	Moderate	78	16.9	16.9	61.0	
	Severe	64	13.9	13.9	74.9	
	ACLF	116	25.1	25.1	100.0	
	Total	462	100.0	100.0		


Mortality ( yes / no) * DILI_severity
Crosstab	
	DILI_severity	
	Mild	Moderate	Severe	
Mortality ( yes / no)	no	Count	162	58	48	
		% within DILI_severity	79.4%	74.4%	75.0%	
	yes	Count	42	20	16	
		% within DILI_severity	20.6%	25.6%	25.0%	
Total	Count	204	78	64	
	% within DILI_severity	100.0%	100.0%	100.0%	

Crosstab	
	DILI_severity	Total	
	ACLF		
Mortality ( yes / no)	no	Count	70	338	
		% within DILI_severity	61.4%	73.5%	
	yes	Count	44	122	
		% within DILI_severity	38.6%	26.5%	
Total	Count	114	460	
	% within DILI_severity	100.0%	100.0%	


Chi-Square Tests	
	Value	df	Asymp. Sig. (2-sided)	
Pearson Chi-Square	12.322a	3	.006	
Likelihood Ratio	11.881	3	.008	
Linear-by-Linear Association	10.822	1	.001	
N of Valid Cases	460			
a. 0 cells (.0%) have expected count less than 5. The minimum expected count is 16.97.
	


age_cat * DILI_severity
Crosstab	
	DILI_severity	Total	
	Mild	Moderate	Severe	ACLF		
age_cat	<=35	Count	46	16	12	28	102	
		% within DILI_severity	22.5%	20.5%	18.8%	24.1%	22.1%	
	36-45	Count	36	14	8	20	78	
		% within DILI_severity	17.6%	17.9%	12.5%	17.2%	16.9%	
	46-55	Count	40	10	12	26	88	
		% within DILI_severity	19.6%	12.8%	18.8%	22.4%	19.0%	
	56-65	Count	40	18	12	30	100	
		% within DILI_severity	19.6%	23.1%	18.8%	25.9%	21.6%	
	>65	Count	42	20	20	12	94	
		% within DILI_severity	20.6%	25.6%	31.3%	10.3%	20.3%	
Total	Count	204	78	64	116	462	
	% within DILI_severity	100.0%	100.0%	100.0%	100.0%	100.0%	


Chi-Square Tests	
	Value	df	Asymp. Sig. (2-sided)	
Pearson Chi-Square	16.015a	12	.191	
Likelihood Ratio	16.888	12	.154	
Linear-by-Linear Association	.302	1	.583	
N of Valid Cases	462			
a. 0 cells (.0%) have expected count less than 5. The minimum expected count is 10.81.
	

Gender * DILI_severity
Crosstab	
	DILI_severity	Total	
	Mild	Moderate	Severe	ACLF		
Gender	Male	Count	102	52	50	62	266	
		% within DILI_severity	50.0%	66.7%	78.1%	53.4%	57.6%	
	Female	Count	102	26	14	54	196	
		% within DILI_severity	50.0%	33.3%	21.9%	46.6%	42.4%	
Total	Count	204	78	64	116	462	
	% within DILI_severity	100.0%	100.0%	100.0%	100.0%	100.0%	

Chi-Square Tests	
	Value	df	Asymp. Sig. (2-sided)	
Pearson Chi-Square	19.306a	3	.000	
Likelihood Ratio	20.222	3	.000	
Linear-by-Linear Association	2.073	1	.150	
N of Valid Cases	462			
a. 0 cells (.0%) have expected count less than 5. The minimum expected count is 27.15.
	


Frequencies


ATT_cat	
	Frequency	Percent	Valid Percent	Cumulative Percent	
Valid	ATT alone	182	39.4	61.7	61.7	
	ATT with combination	113	24.5	38.3	100.0	
	Total	295	63.9	100.0		
Missing	System	167	36.1			
Total	462	100.0			


Att_comb	
	Frequency	Percent	Valid Percent	Cumulative Percent	
Valid	ATT	182	39.4	61.7	61.7	
	att/NSAID	26	5.6	8.8	70.5	
	ATT/antibiotic	83	18.0	28.1	98.6	
	ATT/epileptic	4	.9	1.4	100.0	
	Total	295	63.9	100.0		
Missing	System	167	36.1			
Total	462	100.0			


T-Test
Group Statistics	
	Mortality ( yes / no)	N	Mean	Std. Deviation	Std. Error Mean	
Age	yes	122	53.38	15.162	1.373	
	no	340	49.76	17.010	.922	


Independent Samples Test	
	Levene's Test for Equality of Variances	
	F	Sig.	
Age	Equal variances assumed	8.731	.003	
	Equal variances not assumed			

Independent Samples Test	
	t-test for Equality of Means	
	t	df	Sig. (2-tailed)	Mean Difference	
Age	Equal variances assumed	2.072	460	.039	3.618	
	Equal variances not assumed	2.188	237.668	.030	3.618	


Gender * Mortality ( yes / no)
Crosstab	
	Mortality ( yes / no)	Total	
	no	yes		
Gender	Male	Count	186	80	266	
		% within Mortality ( yes / no)	54.7%	65.6%	57.6%	
	Female	Count	154	42	196	
		% within Mortality ( yes / no)	45.3%	34.4%	42.4%	
Total	Count	340	122	462	
	% within Mortality ( yes / no)	100.0%	100.0%	100.0%	


Chi-Square Tests	
	Value	df	Asymp. Sig. (2-sided)	Exact Sig. (2-sided)	Exact Sig. (1-sided)	
Pearson Chi-Square	4.341a	1	.037			
Continuity Correctionb	3.908	1	.048			
Likelihood Ratio	4.404	1	.036			
Fisher's Exact Test				.042	.023	
Linear-by-Linear Association	4.332	1	.037			
N of Valid Cases	462					
a. 0 cells (.0%) have expected count less than 5. The minimum expected count is 51.76.
b. Computed only for a 2x2 table
	

DM * Mortality ( yes / no)
Crosstab	
	Mortality ( yes / no)	Total	
	no	yes		
DM	YES	Count	72	30	102	
		% within Mortality ( yes / no)	21.2%	24.6%	22.1%	
	NO	Count	268	92	360	
		% within Mortality ( yes / no)	78.8%	75.4%	77.9%	
Total	Count	340	122	462	
	% within Mortality ( yes / no)	100.0%	100.0%	100.0%	


Chi-Square Tests	
	Value	df	Asymp. Sig. (2-sided)	Exact Sig. (2-sided)	Exact Sig. (1-sided)	
Pearson Chi-Square	.608a	1	.435			
Continuity Correctionb	.426	1	.514			
Likelihood Ratio	.598	1	.439			
Fisher's Exact Test				.447	.255	
Linear-by-Linear Association	.607	1	.436			
N of Valid Cases	462					
a. 0 cells (.0%) have expected count less than 5. The minimum expected count is 26.94.
b. Computed only for a 2x2 table
	


Dyslipidemia * Mortality ( yes / no)
Crosstab	
	Mortality ( yes / no)	Total	
	no	yes		
Dyslipidemia	yes	Count	110	50	160	
		% within Mortality ( yes / no)	32.4%	41.0%	34.6%	
	no	Count	230	72	302	
		% within Mortality ( yes / no)	67.6%	59.0%	65.4%	
Total	Count	340	122	462	
	% within Mortality ( yes / no)	100.0%	100.0%	100.0%	


Chi-Square Tests	
	Value	df	Asymp. Sig. (2-sided)	Exact Sig. (2-sided)	Exact Sig. (1-sided)	
Pearson Chi-Square	2.954a	1	.086			
Continuity Correctionb	2.585	1	.108			
Likelihood Ratio	2.909	1	.088			
Fisher's Exact Test				.096	.055	
Linear-by-Linear Association	2.948	1	.086			
N of Valid Cases	462					
a. 0 cells (.0%) have expected count less than 5. The minimum expected count is 42.25.
b. Computed only for a 2x2 table
	
att * Mortality ( yes / no)
Crosstab	
	Mortality ( yes / no)	Total	
	no	yes		
att	yes	Count	224	70	294	
		% within Mortality ( yes / no)	66.3%	57.4%	63.9%	
	no	Count	114	52	166	
		% within Mortality ( yes / no)	33.7%	42.6%	36.1%	
Total	Count	338	122	460	
	% within Mortality ( yes / no)	100.0%	100.0%	100.0%	


Chi-Square Tests	
	Value	df	Asymp. Sig. (2-sided)	Exact Sig. (2-sided)	Exact Sig. (1-sided)	
Pearson Chi-Square	3.075a	1	.079			
Continuity Correctionb	2.702	1	.100			
Likelihood Ratio	3.033	1	.082			
Fisher's Exact Test				.099	.051	
Linear-by-Linear Association	3.069	1	.080			
N of Valid Cases	460					
a. 0 cells (.0%) have expected count less than 5. The minimum expected count is 44.03.
b. Computed only for a 2x2 table
	
antibiotics * Mortality ( yes / no)
Crosstab	
	Mortality ( yes / no)	Total	
	no	yes		
antibiotics	yes	Count	20	8	28	
		% within Mortality ( yes / no)	5.9%	6.6%	6.1%	
	no	Count	320	114	434	
		% within Mortality ( yes / no)	94.1%	93.4%	93.9%	
Total	Count	340	122	462	
	% within Mortality ( yes / no)	100.0%	100.0%	100.0%	

Chi-Square Tests	
	Value	df	Asymp. Sig. (2-sided)	Exact Sig. (2-sided)	Exact Sig. (1-sided)	
Pearson Chi-Square	.072a	1	.789			
Continuity Correctionb	.002	1	.963			
Likelihood Ratio	.071	1	.790			
Fisher's Exact Test				.826	.469	
Linear-by-Linear Association	.072	1	.789			
N of Valid Cases	462					
a. 0 cells (.0%) have expected count less than 5. The minimum expected count is 7.39.
b. Computed only for a 2x2 table
	

antiepileptics * Mortality ( yes / no)
Crosstab	
	Mortality ( yes / no)	Total	
	no	yes		
antiepileptics	yes	Count	8	0	8	
		% within Mortality ( yes / no)	2.4%	.0%	1.7%	
	no	Count	332	122	454	
		% within Mortality ( yes / no)	97.6%	100.0%	98.3%	
Total	Count	340	122	462	
	% within Mortality ( yes / no)	100.0%	100.0%	100.0%	

Chi-Square Tests	
	Value	df	Asymp. Sig. (2-sided)	Exact Sig. (2-sided)	Exact Sig. (1-sided)	
Pearson Chi-Square	2.921a	1	.087			
Continuity Correctionb	1.702	1	.192			
Likelihood Ratio	4.956	1	.026			
Fisher's Exact Test				.117	.084	
Linear-by-Linear Association	2.915	1	.088			
N of Valid Cases	462					
a. 1 cells (25.0%) have expected count less than 5. The minimum expected count is 2.11.
b. Computed only for a 2x2 table
	
antifungal * Mortality ( yes / no)
Crosstab	
	Mortality ( yes / no)	Total	
	no	yes		
antifungal	yes	Count	16	10	26	
		% within Mortality ( yes / no)	4.7%	8.2%	5.6%	
	no	Count	324	112	436	
		% within Mortality ( yes / no)	95.3%	91.8%	94.4%	
Total	Count	340	122	462	
	% within Mortality ( yes / no)	100.0%	100.0%	100.0%	


Chi-Square Tests	
	Value	df	Asymp. Sig. (2-sided)	Exact Sig. (2-sided)	Exact Sig. (1-sided)	
Pearson Chi-Square	2.060a	1	.151			
Continuity Correctionb	1.455	1	.228			
Likelihood Ratio	1.913	1	.167			
Fisher's Exact Test				.170	.116	
Linear-by-Linear Association	2.056	1	.152			
N of Valid Cases	462					
a. 0 cells (.0%) have expected count less than 5. The minimum expected count is 6.87.
b. Computed only for a 2x2 table
	

chemoname * Mortality ( yes / no)
Crosstab	
	Mortality ( yes / no)	Total	
	no	yes		
chemoname	0	Count	334	112	446	
		% within Mortality ( yes / no)	98.2%	91.8%	96.5%	
	duanorubicin/cytarabine	Count	0	2	2	
		% within Mortality ( yes / no)	.0%	1.6%	.4%	
	rchop	Count	2	2	4	
		% within Mortality ( yes / no)	.6%	1.6%	.9%	
	mtx	Count	4	6	10	
		% within Mortality ( yes / no)	1.2%	4.9%	2.2%	
Total	Count	340	122	462	
	% within Mortality ( yes / no)	100.0%	100.0%	100.0%	


Chi-Square Tests	
	Value	df	Asymp. Sig. (2-sided)	
Pearson Chi-Square	12.911a	3	.005	
Likelihood Ratio	11.694	3	.009	
Linear-by-Linear Association	8.869	1	.003	
N of Valid Cases	462			
a. 5 cells (62.5%) have expected count less than 5. The minimum expected count is .53.
	

hakimiherbel * Mortality ( yes / no)
Crosstab	
	Mortality ( yes / no)	Total	
	no	yes		
hakimiherbel	0	Count	2	0	2	
		% within Mortality ( yes / no)	.6%	.0%	.4%	
	yes	Count	32	10	42	
		% within Mortality ( yes / no)	9.4%	8.2%	9.1%	
	no	Count	306	112	418	
		% within Mortality ( yes / no)	90.0%	91.8%	90.5%	
Total	Count	340	122	462	
	% within Mortality ( yes / no)	100.0%	100.0%	100.0%	


Chi-Square Tests	
	Value	df	Asymp. Sig. (2-sided)	
Pearson Chi-Square	.896a	2	.639	
Likelihood Ratio	1.408	2	.495	
Linear-by-Linear Association	.521	1	.470	
N of Valid Cases	462			
a. 2 cells (33.3%) have expected count less than 5. The minimum expected count is .53.
	


antimalarial * Mortality ( yes / no)
Crosstab	
	Mortality ( yes / no)	Total	
	no	yes		
antimalarial	yes	Count	4	6	10	
		% within Mortality ( yes / no)	1.4%	5.6%	2.5%	
	no	Count	288	102	390	
		% within Mortality ( yes / no)	98.6%	94.4%	97.5%	
Total	Count	292	108	400	
	% within Mortality ( yes / no)	100.0%	100.0%	100.0%	


Chi-Square Tests	
	Value	df	Asymp. Sig. (2-sided)	Exact Sig. (2-sided)	Exact Sig. (1-sided)	
Pearson Chi-Square	5.667a	1	.017			
Continuity Correctionb	4.080	1	.043			
Likelihood Ratio	4.912	1	.027			
Fisher's Exact Test				.027	.027	
Linear-by-Linear Association	5.653	1	.017			
N of Valid Cases	400					
a. 1 cells (25.0%) have expected count less than 5. The minimum expected count is 2.70.
b. Computed only for a 2x2 table
	


digoxin * Mortality ( yes / no)
Crosstab	
	Mortality ( yes / no)	Total	
	no	yes		
digoxin	yes	Count	12	0	12	
		% within Mortality ( yes / no)	4.2%	.0%	3.1%	
	no	Count	272	100	372	
		% within Mortality ( yes / no)	95.8%	100.0%	96.9%	
Total	Count	284	100	384	
	% within Mortality ( yes / no)	100.0%	100.0%	100.0%	


Chi-Square Tests	
	Value	df	Asymp. Sig. (2-sided)	Exact Sig. (2-sided)	Exact Sig. (1-sided)	
Pearson Chi-Square	4.362a	1	.037			
Continuity Correctionb	3.078	1	.079			
Likelihood Ratio	7.375	1	.007			
Fisher's Exact Test				.042	.025	
Linear-by-Linear Association	4.350	1	.037			
N of Valid Cases	384					
a. 1 cells (25.0%) have expected count less than 5. The minimum expected count is 3.13.
b. Computed only for a 2x2 table
	


antidepressants * Mortality ( yes / no)
Crosstab	
	Mortality ( yes / no)	Total	
	no	yes		
antidepressants	yes	Count	14	2	16	
		% within Mortality ( yes / no)	4.9%	2.0%	4.2%	
	no	Count	270	98	368	
		% within Mortality ( yes / no)	95.1%	98.0%	95.8%	
Total	Count	284	100	384	
	% within Mortality ( yes / no)	100.0%	100.0%	100.0%	

Chi-Square Tests	
	Value	df	Asymp. Sig. (2-sided)	Exact Sig. (2-sided)	Exact Sig. (1-sided)	
Pearson Chi-Square	1.590a	1	.207			
Continuity Correctionb	.941	1	.332			
Likelihood Ratio	1.838	1	.175			
Fisher's Exact Test				.258	.167	
Linear-by-Linear Association	1.585	1	.208			
N of Valid Cases	384					
a. 1 cells (25.0%) have expected count less than 5. The minimum expected count is 4.17.
b. Computed only for a 2x2 table
	
ams * Mortality ( yes / no)
Crosstab	
	Mortality ( yes / no)	Total	
	no	yes		
ams	yes	Count	54	46	100	
		% within Mortality ( yes / no)	15.9%	37.7%	21.6%	
	no	Count	286	76	362	
		% within Mortality ( yes / no)	84.1%	62.3%	78.4%	
Total	Count	340	122	462	
	% within Mortality ( yes / no)	100.0%	100.0%	100.0%	

Chi-Square Tests	
	Value	df	Asymp. Sig. (2-sided)	Exact Sig. (2-sided)	Exact Sig. (1-sided)	
Pearson Chi-Square	25.211a	1	.000			
Continuity Correctionb	23.940	1	.000			
Likelihood Ratio	23.357	1	.000			
Fisher's Exact Test				.000	.000	
Linear-by-Linear Association	25.156	1	.000			
N of Valid Cases	462					
a. 0 cells (.0%) have expected count less than 5. The minimum expected count is 26.41.
b. Computed only for a 2x2 table
	


jaundice * Mortality ( yes / no)
Crosstab	
	Mortality ( yes / no)	Total	
	no	yes		
jaundice	yes	Count	176	74	250	
		% within Mortality ( yes / no)	51.8%	60.7%	54.1%	
	no	Count	164	48	212	
		% within Mortality ( yes / no)	48.2%	39.3%	45.9%	
Total	Count	340	122	462	
	% within Mortality ( yes / no)	100.0%	100.0%	100.0%	

Chi-Square Tests	
	Value	df	Asymp. Sig. (2-sided)	Exact Sig. (2-sided)	Exact Sig. (1-sided)	
Pearson Chi-Square	2.858a	1	.091			
Continuity Correctionb	2.511	1	.113			
Likelihood Ratio	2.878	1	.090			
Fisher's Exact Test				.112	.056	
Linear-by-Linear Association	2.852	1	.091			
N of Valid Cases	462					
a. 0 cells (.0%) have expected count less than 5. The minimum expected count is 55.98.
b. Computed only for a 2x2 table
	

pruritis * Mortality ( yes / no)
Crosstab	
	Mortality ( yes / no)	Total	
	no	yes		
pruritis	yes	Count	186	78	264	
		% within Mortality ( yes / no)	54.7%	63.9%	57.1%	
	no	Count	154	44	198	
		% within Mortality ( yes / no)	45.3%	36.1%	42.9%	
Total	Count	340	122	462	
	% within Mortality ( yes / no)	100.0%	100.0%	100.0%	


Chi-Square Tests	
	Value	df	Asymp. Sig. (2-sided)	Exact Sig. (2-sided)	Exact Sig. (1-sided)	
Pearson Chi-Square	3.122a	1	.077			
Continuity Correctionb	2.757	1	.097			
Likelihood Ratio	3.157	1	.076			
Fisher's Exact Test				.088	.048	
Linear-by-Linear Association	3.116	1	.078			
N of Valid Cases	462					
a. 0 cells (.0%) have expected count less than 5. The minimum expected count is 52.29.
b. Computed only for a 2x2 table
	

abdpain * Mortality ( yes / no)
Crosstab	
	Mortality ( yes / no)	Total	
	no	yes		
abdpain	yes	Count	328	120	448	
		% within Mortality ( yes / no)	96.5%	98.4%	97.0%	
	no	Count	12	2	14	
		% within Mortality ( yes / no)	3.5%	1.6%	3.0%	
Total	Count	340	122	462	
	% within Mortality ( yes / no)	100.0%	100.0%	100.0%	


Chi-Square Tests	
	Value	df	Asymp. Sig. (2-sided)	Exact Sig. (2-sided)	Exact Sig. (1-sided)	
Pearson Chi-Square	1.092a	1	.296			
Continuity Correctionb	.543	1	.461			
Likelihood Ratio	1.235	1	.266			
Fisher's Exact Test				.373	.238	
Linear-by-Linear Association	1.089	1	.297			
N of Valid Cases	462					
a. 1 cells (25.0%) have expected count less than 5. The minimum expected count is 3.70.
b. Computed only for a 2x2 table
	


NAC( acetylcystiene) y/N * Mortality ( yes / no)
Crosstab	
	Mortality ( yes / no)	Total	
	no	yes		
NAC( acetylcystiene) y/N	no	Count	284	84	368	
		% within Mortality ( yes / no)	84.0%	68.9%	80.0%	
	yes	Count	54	38	92	
		% within Mortality ( yes / no)	16.0%	31.1%	20.0%	
Total	Count	338	122	460	
	% within Mortality ( yes / no)	100.0%	100.0%	100.0%	


Chi-Square Tests	
	Value	df	Asymp. Sig. (2-sided)	Exact Sig. (2-sided)	Exact Sig. (1-sided)	
Pearson Chi-Square	12.896a	1	.000			
Continuity Correctionb	11.965	1	.001			
Likelihood Ratio	12.071	1	.001			
Fisher's Exact Test				.001	.000	
Linear-by-Linear Association	12.867	1	.000			
N of Valid Cases	460					
a. 0 cells (.0%) have expected count less than 5. The minimum expected count is 24.40.
b. Computed only for a 2x2 table
	

intubation * Mortality ( yes / no)
Crosstab	
	Mortality ( yes / no)	Total	
	no	yes		
intubation	yes	Count	18	32	50	
		% within Mortality ( yes / no)	6.9%	38.1%	14.5%	
	no	Count	242	52	294	
		% within Mortality ( yes / no)	93.1%	61.9%	85.5%	
Total	Count	260	84	344	
	% within Mortality ( yes / no)	100.0%	100.0%	100.0%	


Chi-Square Tests	
	Value	df	Asymp. Sig. (2-sided)	Exact Sig. (2-sided)	Exact Sig. (1-sided)	
Pearson Chi-Square	49.662a	1	.000			
Continuity Correctionb	47.185	1	.000			
Likelihood Ratio	42.718	1	.000			
Fisher's Exact Test				.000	.000	
Linear-by-Linear Association	49.518	1	.000			
N of Valid Cases	344					
a. 0 cells (.0%) have expected count less than 5. The minimum expected count is 12.21.
b. Computed only for a 2x2 table
	
Hospital stay
Group Statistics	
	Mortality ( yes / no)	N	Mean	Std. Deviation	Std. Error Mean	
Duration of hospital stay	yes	122	10.75	10.907	.987	
	no	340	6.72	6.556	.356	


Independent Samples Test	
	Levene's Test for Equality of Variances	
	F	Sig.	
Duration of hospital stay	Equal variances assumed	41.480	.000	
	Equal variances not assumed			

Independent Samples Test	
	t-test for Equality of Means	
	t	df	Sig. (2-tailed)	
Duration of hospital stay	Equal variances assumed	4.813	460	.000	
	Equal variances not assumed	3.840	153.489	.000	


T-Test
Group Statistics	
	Mortality ( yes / no)	N	Mean	Std. Deviation	Std. Error Mean	
TB	yes	122	6.916	12.8741	1.1656	
	no	340	4.815	7.0273	.3811	
inr	yes	116	1.9310	1.35777	.12607	
	no	312	1.4576	.65459	.03706	
db	yes	120	4.5767	7.57553	.69155	
	no	340	3.7706	6.15075	.33357	
ib	yes	120	2.5333	5.76517	.52629	
	no	340	1.0818	1.43922	.07805	
GGT	yes	122	144.10	216.292	19.582	
	no	340	142.44	129.527	7.025	
SGPT	yes	122	381.18	753.145	68.187	
	no	340	411.98	993.005	53.853	
AP 	yes	122	202.75	183.185	16.585	
	no	340	164.79	121.118	6.569	
sgot	yes	116	408.1724	789.80112	73.33119	
	no	324	435.7247	1011.73111	56.20728	


Independent Samples Test	
	Levene's Test for Equality of Variances	
	F	Sig.	
TB	Equal variances assumed	9.786	.002	
	Equal variances not assumed			
inr	Equal variances assumed	47.623	.000	
	Equal variances not assumed			
db	Equal variances assumed	1.040	.308	
	Equal variances not assumed			
ib	Equal variances assumed	55.274	.000	
	Equal variances not assumed			
GGT	Equal variances assumed	.342	.559	
	Equal variances not assumed			
SGPT	Equal variances assumed	.045	.831	
	Equal variances not assumed			
AP 	Equal variances assumed	20.800	.000	
	Equal variances not assumed			
sgot	Equal variances assumed	.006	.937	
	Equal variances not assumed			

Independent Samples Test	
	t-test for Equality of Means	
	t	df	Sig. (2-tailed)	Mean Difference	
TB	Equal variances assumed	2.227	460	.026	2.1017	
	Equal variances not assumed	1.714	147.653	.089	2.1017	
inr	Equal variances assumed	4.836	426	.000	.47347	
	Equal variances not assumed	3.603	135.360	.000	.47347	
db	Equal variances assumed	1.159	458	.247	.80608	
	Equal variances not assumed	1.050	177.444	.295	.80608	
ib	Equal variances assumed	4.287	458	.000	1.45157	
	Equal variances not assumed	2.728	124.271	.007	1.45157	
GGT	Equal variances assumed	.100	460	.920	1.657	
	Equal variances not assumed	.080	153.239	.937	1.657	
SGPT	Equal variances assumed	-.312	460	.755	-30.796	
	Equal variances not assumed	-.354	280.129	.723	-30.796	
AP 	Equal variances assumed	2.567	460	.011	37.960	
	Equal variances not assumed	2.128	160.528	.035	37.960	
sgot	Equal variances assumed	-.266	438	.791	-27.55228	
	Equal variances not assumed	-.298	258.100	.766	-27.55228	


Gender
Gender * h_stay Crosstabulation	
	h_stay	Total	
	<5 days	>5 days		
Gender	Male	Count	154	112	266	
		% within h_stay	62.1%	52.3%	57.6%	
	Female	Count	94	102	196	
		% within h_stay	37.9%	47.7%	42.4%	
Total	Count	248	214	462	
	% within h_stay	100.0%	100.0%	100.0%	


Chi-Square Tests	
	Value	df	Asymp. Sig. (2-sided)	Exact Sig. (2-sided)	Exact Sig. (1-sided)	
Pearson Chi-Square	4.480a	1	.034			
Continuity Correctionb	4.090	1	.043			
Likelihood Ratio	4.482	1	.034			
Fisher's Exact Test				.038	.022	
Linear-by-Linear Association	4.471	1	.034			
N of Valid Cases	462					
a. 0 cells (.0%) have expected count less than 5. The minimum expected count is 90.79.
b. Computed only for a 2x2 table
	
T-Test
Group Statistics	
	h_stay	N	Mean	Std. Deviation	Std. Error Mean	
Age	>5 days	214	51.33	16.423	1.123	
	<5 days	248	50.19	16.772	1.065	


Independent Samples Test	
	Levene's Test for Equality of Variances	
	F	Sig.	
Age	Equal variances assumed	.001	.975	
	Equal variances not assumed			

Independent Samples Test	
	t-test for Equality of Means	
	t	df	Sig. (2-tailed)	Mean Difference	
Age	Equal variances assumed	.737	460	.462	1.142	
	Equal variances not assumed	.738	452.711	.461	1.142	


DM * h_stay
Crosstab	
	h_stay	Total	
	<5 days	>5 days		
DM	YES	Count	58	44	102	
		% within h_stay	23.4%	20.6%	22.1%	
	NO	Count	190	170	360	
		% within h_stay	76.6%	79.4%	77.9%	
Total	Count	248	214	462	
	% within h_stay	100.0%	100.0%	100.0%	


Chi-Square Tests	
	Value	df	Asymp. Sig. (2-sided)	Exact Sig. (2-sided)	Exact Sig. (1-sided)	
Pearson Chi-Square	.533a	1	.465			
Continuity Correctionb	.382	1	.537			
Likelihood Ratio	.535	1	.465			
Fisher's Exact Test				.501	.269	
Linear-by-Linear Association	.532	1	.466			
N of Valid Cases	462					
a. 0 cells (.0%) have expected count less than 5. The minimum expected count is 47.25.
b. Computed only for a 2x2 table
	

Dyslipidemia * h_stay
Crosstab	
	h_stay	Total	
	<5 days	>5 days		
Dyslipidemia	yes	Count	80	80	160	
		% within h_stay	32.3%	37.4%	34.6%	
	no	Count	168	134	302	
		% within h_stay	67.7%	62.6%	65.4%	
Total	Count	248	214	462	
	% within h_stay	100.0%	100.0%	100.0%	


Chi-Square Tests	
	Value	df	Asymp. Sig. (2-sided)	Exact Sig. (2-sided)	Exact Sig. (1-sided)	
Pearson Chi-Square	1.333a	1	.248			
Continuity Correctionb	1.116	1	.291			
Likelihood Ratio	1.332	1	.249			
Fisher's Exact Test				.281	.145	
Linear-by-Linear Association	1.330	1	.249			
N of Valid Cases	462					
a. 0 cells (.0%) have expected count less than 5. The minimum expected count is 74.11.
b. Computed only for a 2x2 table
	


att * h_stay


Crosstab	
	h_stay	Total	
	<5 days	>5 days		
att	yes	Count	180	114	294	
		% within h_stay	73.2%	53.3%	63.9%	
	no	Count	66	100	166	
		% within h_stay	26.8%	46.7%	36.1%	
Total	Count	246	214	460	
	% within h_stay	100.0%	100.0%	100.0%	


Chi-Square Tests	
	Value	df	Asymp. Sig. (2-sided)	Exact Sig. (2-sided)	Exact Sig. (1-sided)	
Pearson Chi-Square	19.649a	1	.000			
Continuity Correctionb	18.796	1	.000			
Likelihood Ratio	19.729	1	.000			
Fisher's Exact Test				.000	.000	
Linear-by-Linear Association	19.606	1	.000			
N of Valid Cases	460					
a. 0 cells (.0%) have expected count less than 5. The minimum expected count is 77.23.
b. Computed only for a 2x2 table
	
antibiotics * h_stay
Crosstab	
	h_stay	Total	
	<5 days	>5 days		
antibiotics	yes	Count	12	16	28	
		% within h_stay	4.8%	7.5%	6.1%	
	no	Count	236	198	434	
		% within h_stay	95.2%	92.5%	93.9%	
Total	Count	248	214	462	
	% within h_stay	100.0%	100.0%	100.0%	


Chi-Square Tests	
	Value	df	Asymp. Sig. (2-sided)	Exact Sig. (2-sided)	Exact Sig. (1-sided)	
Pearson Chi-Square	1.404a	1	.236			
Continuity Correctionb	.979	1	.322			
Likelihood Ratio	1.400	1	.237			
Fisher's Exact Test				.248	.161	
Linear-by-Linear Association	1.401	1	.237			
N of Valid Cases	462					
a. 0 cells (.0%) have expected count less than 5. The minimum expected count is 12.97.
b. Computed only for a 2x2 table
	
antiepileptics * h_stay
Crosstab	
	h_stay	Total	
	<5 days	>5 days		
antiepileptics	yes	Count	6	2	8	
		% within h_stay	2.4%	.9%	1.7%	
	no	Count	242	212	454	
		% within h_stay	97.6%	99.1%	98.3%	
Total	Count	248	214	462	
	% within h_stay	100.0%	100.0%	100.0%	


Chi-Square Tests	
	Value	df	Asymp. Sig. (2-sided)	Exact Sig. (2-sided)	Exact Sig. (1-sided)	
Pearson Chi-Square	1.488a	1	.222			
Continuity Correctionb	.744	1	.389			
Likelihood Ratio	1.572	1	.210			
Fisher's Exact Test				.296	.196	
Linear-by-Linear Association	1.485	1	.223			
N of Valid Cases	462					
a. 2 cells (50.0%) have expected count less than 5. The minimum expected count is 3.71.
b. Computed only for a 2x2 table
	


antifungal * h_stay
Crosstab	
	h_stay	Total	
	<5 days	>5 days		
antifungal	yes	Count	12	14	26	
		% within h_stay	4.8%	6.5%	5.6%	
	no	Count	236	200	436	
		% within h_stay	95.2%	93.5%	94.4%	
Total	Count	248	214	462	
	% within h_stay	100.0%	100.0%	100.0%	


Chi-Square Tests	
	Value	df	Asymp. Sig. (2-sided)	Exact Sig. (2-sided)	Exact Sig. (1-sided)	
Pearson Chi-Square	.628a	1	.428			
Continuity Correctionb	.348	1	.555			
Likelihood Ratio	.625	1	.429			
Fisher's Exact Test				.544	.277	
Linear-by-Linear Association	.626	1	.429			
N of Valid Cases	462					
a. 0 cells (.0%) have expected count less than 5. The minimum expected count is 12.04.
b. Computed only for a 2x2 table
	
amiodarone * h_stay
Crosstab	
	h_stay	Total	
	<5 days	>5 days		
amiodarone	0	Count	2	0	2	
		% within h_stay	.8%	.0%	.4%	
	yes	Count	10	14	24	
		% within h_stay	4.0%	6.5%	5.2%	
	yes	Count	236	200	436	
		% within h_stay	95.2%	93.5%	94.4%	
Total	Count	248	214	462	
	% within h_stay	100.0%	100.0%	100.0%	

Chi-Square Tests	
	Value	df	Asymp. Sig. (2-sided)	
Pearson Chi-Square	3.154a	2	.207	
Likelihood Ratio	3.914	2	.141	
Linear-by-Linear Association	.141	1	.708	
N of Valid Cases	462			
a. 2 cells (33.3%) have expected count less than 5. The minimum expected count is .93.
	
lipiget * h_stay
Crosstab	
	h_stay	Total	
	<5 days	>5 days		
lipiget	0	Count	0	2	2	
		% within h_stay	.0%	.9%	.4%	
	yes	Count	18	14	32	
		% within h_stay	7.3%	6.5%	6.9%	
	no	Count	230	198	428	
		% within h_stay	92.7%	92.5%	92.6%	
Total	Count	248	214	462	
	% within h_stay	100.0%	100.0%	100.0%	


Chi-Square Tests	
	Value	df	Asymp. Sig. (2-sided)	
Pearson Chi-Square	2.403a	2	.301	
Likelihood Ratio	3.164	2	.206	
Linear-by-Linear Association	.189	1	.663	
N of Valid Cases	462			
a. 2 cells (33.3%) have expected count less than 5. The minimum expected count is .93.
	

chemo * h_stay
Crosstab	
	h_stay	Total	
	<5 days	>5 days		
chemo	0	Count	4	0	4	
		% within h_stay	1.6%	.0%	.9%	
	yes	Count	6	10	16	
		% within h_stay	2.4%	4.7%	3.5%	
	no	Count	238	204	442	
		% within h_stay	96.0%	95.3%	95.7%	
Total	Count	248	214	462	
	% within h_stay	100.0%	100.0%	100.0%	


Chi-Square Tests	
	Value	df	Asymp. Sig. (2-sided)	
Pearson Chi-Square	5.141a	2	.076	
Likelihood Ratio	6.669	2	.036	
Linear-by-Linear Association	.163	1	.687	
N of Valid Cases	462			
a. 2 cells (33.3%) have expected count less than 5. The minimum expected count is 1.85.
	

hakimiherbel * h_stay
Crosstab	
	h_stay	Total	
	<5 days	>5 days		
hakimiherbel	0	Count	0	2	2	
		% within h_stay	.0%	.9%	.4%	
	yes	Count	22	20	42	
		% within h_stay	8.9%	9.3%	9.1%	
	no	Count	226	192	418	
		% within h_stay	91.1%	89.7%	90.5%	
Total	Count	248	214	462	
	% within h_stay	100.0%	100.0%	100.0%	


Chi-Square Tests	
	Value	df	Asymp. Sig. (2-sided)	
Pearson Chi-Square	2.371a	2	.306	
Likelihood Ratio	3.132	2	.209	
Linear-by-Linear Association	.641	1	.423	
N of Valid Cases	462			
a. 2 cells (33.3%) have expected count less than 5. The minimum expected count is .93.
	

antimalarial * h_stay
Crosstab	
	h_stay	Total	
	<5 days	>5 days		
antimalarial	yes	Count	2	8	10	
		% within h_stay	.9%	4.5%	2.5%	
	no	Count	220	170	390	
		% within h_stay	99.1%	95.5%	97.5%	
Total	Count	222	178	400	
	% within h_stay	100.0%	100.0%	100.0%	


Chi-Square Tests	
	Value	df	Asymp. Sig. (2-sided)	Exact Sig. (2-sided)	Exact Sig. (1-sided)	
Pearson Chi-Square	5.234a	1	.022			
Continuity Correctionb	3.863	1	.049			
Likelihood Ratio	5.433	1	.020			
Fisher's Exact Test				.027	.024	
Linear-by-Linear Association	5.220	1	.022			
N of Valid Cases	400					
a. 1 cells (25.0%) have expected count less than 5. The minimum expected count is 4.45.
b. Computed only for a 2x2 table
	

digoxin * h_stay
Crosstab	
	h_stay	Total	
	<5 days	>5 days		
digoxin	yes	Count	6	6	12	
		% within h_stay	2.7%	3.7%	3.1%	
	no	Count	214	158	372	
		% within h_stay	97.3%	96.3%	96.9%	
Total	Count	220	164	384	
	% within h_stay	100.0%	100.0%	100.0%	


Chi-Square Tests	
	Value	df	Asymp. Sig. (2-sided)	Exact Sig. (2-sided)	Exact Sig. (1-sided)	
Pearson Chi-Square	.269a	1	.604			
Continuity Correctionb	.049	1	.824			
Likelihood Ratio	.266	1	.606			
Fisher's Exact Test				.768	.407	
Linear-by-Linear Association	.268	1	.604			
N of Valid Cases	384					
a. 0 cells (.0%) have expected count less than 5. The minimum expected count is 5.13.
b. Computed only for a 2x2 table
	

Mortality ( yes / no) * h_stay
Crosstab	
	h_stay	Total	
	<5 days	>5 days		
Mortality ( yes / no)	no	Count	194	146	340	
		% within h_stay	78.2%	68.2%	73.6%	
	yes	Count	54	68	122	
		% within h_stay	21.8%	31.8%	26.4%	
Total	Count	248	214	462	
	% within h_stay	100.0%	100.0%	100.0%	


Chi-Square Tests	
	Value	df	Asymp. Sig. (2-sided)	Exact Sig. (2-sided)	Exact Sig. (1-sided)	
Pearson Chi-Square	5.913a	1	.015			
Continuity Correctionb	5.409	1	.020			
Likelihood Ratio	5.905	1	.015			
Fisher's Exact Test				.020	.010	
Linear-by-Linear Association	5.900	1	.015			
N of Valid Cases	462					
a. 0 cells (.0%) have expected count less than 5. The minimum expected count is 56.51.
b. Computed only for a 2x2 table
	
NAC( acetylcystiene) y/N * h_stay
Crosstab	
	h_stay	Total	
	<5 days	>5 days		
NAC( acetylcystiene) y/N	no	Count	206	162	368	
		% within h_stay	83.1%	76.4%	80.0%	
	yes	Count	42	50	92	
		% within h_stay	16.9%	23.6%	20.0%	
Total	Count	248	212	460	
	% within h_stay	100.0%	100.0%	100.0%	


Chi-Square Tests	
	Value	df	Asymp. Sig. (2-sided)	Exact Sig. (2-sided)	Exact Sig. (1-sided)	
Pearson Chi-Square	3.158a	1	.076			
Continuity Correctionb	2.757	1	.097			
Likelihood Ratio	3.150	1	.076			
Fisher's Exact Test				.080	.049	
Linear-by-Linear Association	3.152	1	.076			
N of Valid Cases	460					
a. 0 cells (.0%) have expected count less than 5. The minimum expected count is 42.40.
b. Computed only for a 2x2 table
	
intubation * h_stay
Crosstab	
	h_stay	Total	
	<5 days	>5 days		
intubation	yes	Count	16	34	50	
		% within h_stay	9.9%	18.7%	14.5%	
	no	Count	146	148	294	
		% within h_stay	90.1%	81.3%	85.5%	
Total	Count	162	182	344	
	% within h_stay	100.0%	100.0%	100.0%	


Chi-Square Tests	
	Value	df	Asymp. Sig. (2-sided)	Exact Sig. (2-sided)	Exact Sig. (1-sided)	
Pearson Chi-Square	5.349a	1	.021			
Continuity Correctionb	4.664	1	.031			
Likelihood Ratio	5.478	1	.019			
Fisher's Exact Test				.022	.015	
Linear-by-Linear Association	5.333	1	.021			
N of Valid Cases	344					
a. 0 cells (.0%) have expected count less than 5. The minimum expected count is 23.55.
b. Computed only for a 2x2 table
	
H/O alcohol * h_stay


Crosstab	
	h_stay	Total	
	<5 days	>5 days		
H/O alcohol	yes	Count	2	4	6	
		% within h_stay	.8%	1.9%	1.3%	
	no	Count	244	210	454	
		% within h_stay	99.2%	98.1%	98.7%	
Total	Count	246	214	460	
	% within h_stay	100.0%	100.0%	100.0%	


Chi-Square Tests	
	Value	df	Asymp. Sig. (2-sided)	Exact Sig. (2-sided)	Exact Sig. (1-sided)	
Pearson Chi-Square	.992a	1	.319			
Continuity Correctionb	.341	1	.559			
Likelihood Ratio	1.000	1	.317			
Fisher's Exact Test				.424	.280	
Linear-by-Linear Association	.989	1	.320			
N of Valid Cases	460					
a. 2 cells (50.0%) have expected count less than 5. The minimum expected count is 2.79.
b. Computed only for a 2x2 table
	
T-Test
Group Statistics	
	h_stay	N	Mean	Std. Deviation	Std. Error Mean	
TB	>5 days	214	6.123	11.2795	.7710	
	<5 days	248	4.719	6.3202	.4013	
inr	>5 days	200	1.5719	.80417	.05686	
	<5 days	228	1.5982	1.01846	.06745	
ib	>5 days	214	1.8439	4.50035	.30764	
	<5 days	246	1.1268	1.38729	.08845	
GGT	>5 days	214	137.17	181.847	12.431	
	<5 days	248	147.81	131.817	8.370	
SGPT	>5 days	214	500.79	1151.464	78.712	
	<5 days	248	320.19	687.767	43.673	
AP 	>5 days	214	174.43	157.806	10.787	
	<5 days	248	175.15	124.984	7.937	
sgot	>5 days	200	525.4340	1166.84557	82.50844	
	<5 days	240	347.6500	731.91079	47.24464	
R ratio( ALT/ALT uln / AP/Ap uln)	>5 days	2	34.0000	.00000a	.00000	
	<5 days	2	38.0000	.00000a	.00000	
a. t cannot be computed because the standard deviations of both groups are 0.
	


Independent Samples Test	
	Levene's Test for Equality of Variances	
	F	Sig.	
TB	Equal variances assumed	14.868	.000	
	Equal variances not assumed			
inr	Equal variances assumed	1.190	.276	
	Equal variances not assumed			
ib	Equal variances assumed	21.255	.000	
	Equal variances not assumed			
GGT	Equal variances assumed	.017	.896	
	Equal variances not assumed			
SGPT	Equal variances assumed	12.013	.001	
	Equal variances not assumed			
AP 	Equal variances assumed	.005	.943	
	Equal variances not assumed			
sgot	Equal variances assumed	9.228	.003	
	Equal variances not assumed			

Independent Samples Test	
	t-test for Equality of Means	
	t	df	Sig. (2-tailed)	Mean Difference	
TB	Equal variances assumed	1.679	460	.094	1.4040	
	Equal variances not assumed	1.615	323.567	.107	1.4040	
inr	Equal variances assumed	-.293	426	.770	-.02626	
	Equal variances not assumed	-.298	421.479	.766	-.02626	
ib	Equal variances assumed	2.373	458	.018	.71710	
	Equal variances not assumed	2.240	248.196	.026	.71710	
GGT	Equal variances assumed	-.726	460	.468	-10.638	
	Equal variances not assumed	-.710	382.186	.478	-10.638	
SGPT	Equal variances assumed	2.078	460	.038	180.609	
	Equal variances not assumed	2.006	336.806	.046	180.609	
AP 	Equal variances assumed	-.055	460	.956	-.723	
	Equal variances not assumed	-.054	403.936	.957	-.723	
sgot	Equal variances assumed	1.946	438	.052	177.78400	
	Equal variances not assumed	1.870	322.060	.062	177.78400	
